# Comorbidities and Factors Associated with Mortality among Children under Five Years Admitted with Severe Acute Malnutrition in the Nutritional Unit of Jinja Regional Referral Hospital, Eastern Uganda

**DOI:** 10.1155/2020/7809412

**Published:** 2020-11-24

**Authors:** Desire Banga, Melvis Baren, Namale Vivian Ssonko, Franck Katembo Sikakulya, Yves Tibamwenda, Claude Banga, Robinson Ssebuufu

**Affiliations:** ^1^Department of Pediatrics and Child Health, Kampala International University, Western Campus, Ishaka, Uganda; ^2^Department of Surgery, Kampala International University, Western Campus, Ishaka, Uganda; ^3^Faculty of Medicine, Université Catholique du Graben, Butembo, Democratic Republic of the Congo; ^4^Department of Internal Medicine, Kampala International University, Western Campus, Ishaka, Uganda

## Abstract

**Background:**

Mortality among children with severe acute malnutrition remains an immense health concern in the hospitals in developing countries, but its attributes are not completely assessed in various hospital settings. The aim of this study was to determine the proportion of mortality, the comorbidities, and factors associated with in-hospital mortality among children under five years of age admitted with severe acute malnutrition at Jinja Regional Referral Hospital, Eastern Uganda.

**Methods:**

This was a hospital-based analytical and descriptive prospective cohort study conducted in the nutritional unit of Jinja Regional Referral Hospital. A total of 338 children and their caretakers who met the criteria were consecutively enrolled into the study. Descriptive statistics were used to each of the independent factors, and comorbidities were subjected to chi-squared test followed by logistic regression analysis to assess its association incidence of mortality among children. All independent variables with *p* values ≤ 0.05 were entered into a multivariate model for factors and comorbidities independently. Factors and comorbidities with *p* values ≤ 0.05 were considered as associates of mortality among children.

**Results:**

Of the 338 children under 5 years of age enrolled, 49 (14.5%) died, although the majority of children were diagnosed with dehydration, 128 (37.9%); pneumonia, 127(37.6%); and malaria, 87(25.7%). Anemia (aRR = 2.9, 95% CI: 1.23-6.62, *p* = 0.01), bacteremia (aRR = 10.0, 95% CI: 3.62-29.01, *p* = 0.01), HIV (aRR = 4.8, 95% CI: 1.42-16.30, *p* < 0.01), TB (aRR = 4.3, 95% CI: 1.28-14.49, *p* < 0.02), and shock (aRR = 60.9, 95% CI: 9.05-410.28, *p* < 0.01) were the comorbidities significantly associated with a likelihood of mortality.

**Conclusions:**

The mortality among children under 5 years of age admitted with severe acute malnutrition is still high (14.5% versus 5%). The comorbidities are significantly associated with mortality. The clinicians are recommended to follow-up closely patients with severe acute malnutrition and to focus on the critical comorbidities identified.

## 1. Introduction

Globally, severe acute malnutrition affects around 16 million children under 5 years. The risk of death is among children with SAM is nine times greater than well-nourished children [1]. SAM is a significant direct or indirect contributing factor in approximately half of the 5.9 million deaths of children aged under 5 years worldwide [1]. SAM remains a major cause of child morbidity and mortality worldwide, and of the 7.6 million deaths among children under 5 years, approximately 35% are due to nutrition-related factors, and 4.4% of deaths have been shown to be specifically attributable to severe wasting [2].

The majority of the cases of SAM occur in developing countries and are related to chronic poverty, lack of education, poor hygiene, limited access to food, and poor diet, resulting in significant barriers to achievement of Sustainable Development Goals [3]. According to UNICEF, WHO, and the World Bank, 14.1 million children under five years in the African region were wasted (4.3 million of them severely) in 2015, and all had wasting rates of 5-10% [1]. Children suffering from wasting have weakened immunity are susceptible to long-term developmental delays, infection, and face an increased risk of death, particularly when wasting is severe [1]. The mortality of children with severe acute malnutrition varies from one region to another, with 21% in Democratic Republic of Congo, 20% in Senegal, and 20% in Uganda [1]. According to UBOS (2016), stunting accounts for 29%, with 4% of children 6–59 months being wasted, that is to say, too thin for height, while 11% of the children were underweight and 4% were obese [4].

In Uganda, more than 30% of the total population faces some level of food insecurity as a result of poverty, high fertility, being landless, and climate-related change [5]. Statistics show that 300,000 children (5% nationally) have acute malnutrition and nearly 120,000 (2%) of them have SAM [4]. SAM is associated with 1.6 million of annual additional morbidity episodes, with 258 million US dollars of economic cost and contributing to 15% of the total child mortalities in Uganda [6]. In addition, malnutrition in Uganda is concentrated in Northern and Eastern regions. Jinja is located in the Eastern region of Uganda which faces lack of access to sufficient food with 5.1% of severe wasting after West Nile which has a prevalence of 5.3% [4]. The nutritional unit of Jinja Regional Referral Hospital has 32 beds with a minimum of 60 admissions per month. This background explains this study assessed the comorbidities and factors associated with in-hospital mortality among children under five years of age admitted with severe acute malnutrition at Jinja Regional Referral Hospital.

Severe acute malnutrition is common in sub-Saharan Africa. The association with mortality and morbidities is scanty despite the ample literature on the prevalence and factors associated with SAM. Information pertaining to the association between risk factors for increased mortality among severely malnourished children during periods of admission remains scanty in Jinja. WHO estimates that 60% of all deaths occurring among children under five years of age in developing countries is attributed to childhood malnutrition [1]. Uganda has high prevalence rates of malnutrition. The prevalence of global stunting is estimated at 39.1%, underweight 22.85, and global wasting 4.1%. SAM is associated with 1.6 million of annual additional morbidities episodes with 258 million US dollars of economic cost and contributing to 15% of the total child death [6]. Uganda has not yet succeeded to reduce under 5 years mortality to at least as low as 25 deaths per 1,000 live births according to Sustainable Development Goals.

In Northern Uganda, a study by Nyeko et al. [7] on treatment outcome among children with severe acute malnutrition in Lacor Hospital revealed a proportion of 11.9% with hypothermia and HIV being the factors strongly associated with mortality. Another study by Nabukeera-Barungi et al. [8] on predictors of mortality in children with severe acute malnutrition in Mulago Hospital in Kampala showed a proportion of 9.8%, with infections being the major contributors of mortality. Although some studies about mortality in children under five years with severe acute malnutrition have been done in Uganda, those studies were conducted in central and northern Uganda, and there is paucity of data on mortality due to SAM in Eastern Uganda, especially in Jinja.

Therefore, this study set out to determine the proportion of mortality, comorbidities, and factors associated with the in-hospital mortality among children who were with SAM in the nutritional unit of JRRH.

## 2. Methodology

### 2.1. Study Design

This was an analytical descriptive prospective cohort study to determine the proportion of mortality, demographic, biological, socioeconomic factors, and comorbidities associated with mortality among children below 5 years admitted with severe acute malnutrition in the nutritional unit of Jinja Regional Referral Hospital.

### 2.2. Study Site

The study was conducted at Jinja RRH located in southeastern Uganda, approximately 87 kilometers east of Kampala, the capital of Uganda. It is a designated internship hospital where medical graduate internship center and has consultants in medicine and surgery. Jinja RRH also provides comprehensive HIV/AIDS services. It is also a KIU Satellite Teaching Hospital aimed at training undergraduates and postgraduate students including postgraduate in Pediatrics. The nutritional unit is in pediatric ward, around 1 km from the main hospital; it has 32 beds and 3 trained nurses assigned for the ward. Patients are categorized into moderate acute malnutrition, severe acute malnutrition, and edematous and nonedematous malnutrition with a minimum of 80 admissions per month.

### 2.3. Study Population

All children under five years of age admitted with SAM in the nutritional unit of JRRH and their caregivers constituted our population. According to the 2014 census data, Jinja RRH serves a population of 471,242 in Jinja District, and the hospital also serves Bugiri, Kamuli, Iganga, Mayuge, Namutumba, Kaliro, Buyende, Luuka, Namiyongo, and Jinja District.

### 2.4. Selection Criteria

This study included all children under five years of age admitted with SAM in the nutritional unit of Jinja Regional Referral Hospital, and their caregivers who consented were included in this study. Children who were referred for further management were excluded from this study. Also, children with preexisting congenital malformations and cerebral palsy were excluded from this study.

### 2.5. Sample Size Estimation

Daniel population proportion formula and modified Daniel's formula were used to estimate the sample size [9].

(1) $$\begin{array}{c} n=Z_{\alpha /2^{2}}MOE^{2}, \end{array}$$

and

*Z*_*α*/2_ is the critical value of the normal distribution at *α*/2 (e.g., for a confidence level of 95%, *α* is 0.05, and the critical value is 1.96); MOE is the margin of error, estimated at 0.05. *P* is the estimated proportion of children with SAM that died in hospital = 0.33 based on a study done in Kenya that reported 33% [10]. *n* = 1.96^2^ × 0.33 (1 − 0.33)/0.05^2^ = 339.7 = 340 children with severe acute malnutrition.

The overall sample size was 340 children under five years of age admitted with SAM.

### 2.6. Sampling Technique

All children under five years of age admitted with SAM in the nutritional unit of Jinja Regional Referral hospital, who met our inclusion criteria, were consecutively enrolled into the study until when the required sample size has been attained.

### 2.7. Data Collection Tools

The following tools will be used to collect data: questionnaires, tape measure, thermometer, pediatric-size stethoscope, stadiometer, infantometer, WHO chart, vacutainers, gloves, syringes, and culture medium.

### 2.8. Study Procedures

#### 2.8.1. Screening for Eligibility

Screening and inclusion were performed upon admission of the child in the pediatric ward as long as they met inclusion criteria. The principal investigator explained the purpose and the process of the study to the patient and/or guardian, and a written consent was obtained.

#### 2.8.2. Demographic Information and Biological Information

Information regarding the place of residence, sex, age and date of birth, and relationship of the child with the caretaker was collected by the principal investigator using the data collection tool. Complaints of diarrhea, vomiting, loss of appetite, fever, cough, and others were also recorded in the data collection tool as well as the child's immunization history, nutrition history, HIV status, and occupation of the caretaker. All information obtained was entered in the data collection tool. The emergency cases were managed first; information from them was collected later.

#### 2.8.3. Clinical Examination

Children underwent fully physical examination and were classified using WHO guideline. Findings on clinical exam were entered in the data collection tool by the principal investigator; they helped to diagnose some comorbidities. All the patients were followed up from admission to the discharge with the clinical care pathway form.

#### 2.8.4. Sample Collection and Laboratory Procedures

Blood sample was collected during insertion of IV cannula for measurement of full blood count, blood glucose, blood slide for malaria, and HIV test and culture. Prior to drawing of blood, the area will be swabbed with cotton dipped in ethyl alcohol 70% and iodine to prevent contamination. Three to four milliliters of blood was drawn for blood culture, determination of blood glucose, full blood count, HIV test and blood slide for malaria, and serum electrolytes. The blood sample for culture was taken and analyzed using Automated Blood Culture System (BACTEC) in the main hospital laboratory of JRRH. These laboratory investigations were done on admission except serum electrolytes that was done during transition phase. For the HIV test, pre- and posttest counseling were done. A first Determine® HIV rapid test was performed and confirmed with a 2nd rapid test StatPak®. In case of discordance, a Unigold® test will be done. Children under 18 months with positive rapid tests will be referred for DNA PCR test in the HIV clinic in the Jinja RRH. Blood samples will be taken off only once at admission.

Full blood count was analyzed using Sysmex® Automated Hematology Analyser at Jinja RRH. Blood glucose was measured using a Freestyle optium glucometer by the principal investigator at admission. Field stains A and B were done for thick blood smears for malaria and examined by the laboratory technician using the microscope in the pediatric ward laboratory. Early morning gastric aspirate was collected in a sterile container for gene expert at admission.

#### 2.8.5. Comorbidities

After getting the history, clinical assessments coupled with laboratory investigations were done to diagnose the comorbidities related to severe acute malnutrition. Those comorbidities were defined according to WHO protocol [11] as follows:

Hypoglycemia by the blood sugar < 3 mmol/liter (54 mg/dl), hypothermia by the axillary temperature below 35 degree Celsius, and when the axillary temperature did not register on a normal temperature, we assumed that the child had hypothermia. All the participants with SAM who had watery diarrhea or reduced urine output were assumed to have some dehydration. However, electrolyte imbalance was defined by the serum electrolytes below or above the normal ranges. Bacteremia was defined by the bacterial growth in the blood sample collected using Automated Blood Culture system. Severe pneumonia was defined by cough or difficulty in breathing with oxygen saturation < 90% or central cyanosis, severe respiratory distress, or signs of pneumonia with a general danger sigh like inability to breastfeed, lethargy, or altered level of consciousness with decreased breath sound, bronchial breath sound, crackles, and pleural rub on auscultation of the chest. Pneumonia was defined by cough with fast breathing for the age, chest in drawing. Severe anemia was defined by hemoglobin below 4 g/dl or 4-6 g/dl in a child with respiratory distress. Shock was defined by lethargy or unconsciousness with cold extremities, capillary refill above 3 seconds, and a weak or fast pulse or a low or immeasurable systolic blood pressure. Confirmed tuberculosis was defined by a positive gene expert.

#### 2.8.6. Patient Management

Children with SAM were managed according to updated WHO guidelines. Prescription of treatments for the participants was done by doctors and clinical officers who were availed with guidelines on treating children with SAM. The role of the principal investigator was limited on recommendations. All children were routinely treated with IV Ampicillin and IV Gentamycin that was modified according to the blood culture and sensitivity results. They were also routinely started on a feeding program with F75 formula feeds. Caretakers took the responsibility for the feeding of the children under the supervision of the hospital nutritionist. Children with diarrhea were routinely given zinc tablets in addition to the routine deworming tablet that was given to all the enrolled patients. Those with some dehydration were given ReSoMal solution while those with septic shock were given IV half strength Darrow's and 5% dextrose solution. All patients were followed up from admission to the discharge according to the WHO protocol.

### 2.9. Data Analysis

The data was entered and cleaned using EPI info version 7 and was exported to STATA 12.0 for further analysis. Sociodemographic was summarized descriptively as frequencies and percentages (categorical variables).

In-hospital mortality was analyzed as frequency and percentage and presented in a pie chart.

Keeping strata constant, sociodemographic, economic, and biological factors associated with in-hospital mortality were assessed using a Generalized Linear Model to obtain relative risk of mortality. Both bivariate and multivariate analysis were carried out. Crude and adjusted relative risk (RR), their corresponding 95% CI and *p* values were reported. Data presentation was done by using tables.

The proportion of comorbidities was assessed for association with in-hospital mortality using frequencies and percentages, and their association with mortality was assessed using logistic regression to obtain the relative risk of mortality. Both bivariate and multivariate analyses were carried out. Data was presented using a table. Statistical significance was considered at *α* ≤ 0.05.

## 3. Results

Overall, 340 participants under 5 years of age with severe acute malnutrition were consecutively enrolled in the study in the nutritional unit of JRRH after an informed consent of their caregivers from July to September 2019. Of the 340 participants, 2 participants were excluded from the study because of refusal to consent. A total of 338 participants were followed up from admission to discharge. A total of 49 participants died, and 289 participants survived.

### 3.1. Proportion of Mortality among Children under 5 Years with SAM in JRRH

**Figure 1:**
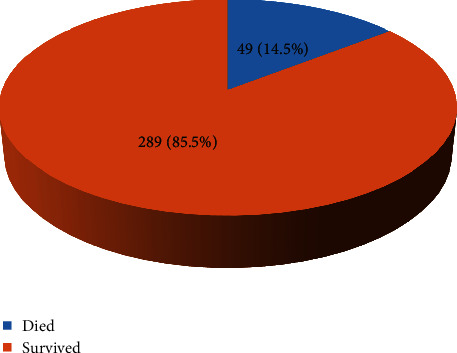
Mortality of children admitted with SAM at Jinja Regional Referral Hospital.

Figure 1 shows that of the 338 children under five years of age admitted with SAM in the nutritional unit of JRRH, a total of 49 children died, giving an overall mortality of 14.5%.

### 3.2. Factors Associated with Mortality among Children below 5 Years Admitted with Severe Acute Malnutrition in the Nutritional Unit of JRRH

Table 1 shows that the sex of the child and their duration of admission (hospital stay) were the independent factors associated with mortality among children under five years admitted at JRRH. Precisely, in the bivariate model, sex of the child, duration of admission, caregiver's highest education level, and caregiver's average monthly income were found to be associated with mortality among children admitted with SAM at JRRH (*p* ≤ 0.05) and were then entered into the multivariate model. Specifically, in the multivariate model, it was found that risk of death was 0.39 time less in males than females (aRR = 0.39, 95% CI: 0.17-0.87, *p* = 0.02). Also, the risk of death among children below 5 years admitted with severe acute malnutrition in the nutritional unit was associated with hospital stay in which duration of admission for 72 hours and above (aRR = 0.04, 95% CI: 0.00-0.40, *p* = 0.01) compared to those children that spent less than 24 hours in hospital.

### 3.3. Comorbidities Associated with in Hospital Mortality among Children under 5 Years with SAM in JRHH

Table 2 shows that in absence of other comorbidities, all the comorbidities except malaria, pneumonia, electrolyte imbalance, and cardiac failure were associated with in-hospital mortality of children under 5 years of age admitted with SAM in the nutritional unit of JRRH (*p* ≤ 0.05). In the multivariate model in which only comorbidities with *p* value ≤ 0.05 were entered, acute diarrhea, bacteremia, HIV, TB, and shock remained significant comorbidities associated with in-hospital mortality. Specifically, children who had acute diarrhea were 0.2 times less likely to experience mortality than those who had no acute diarrhea (aRR = 0.2, 95% CI: 0.04-0.88, *p* = 0.03). Children who had anemia were 2.9 times more likely to die than those who had no anemia (aRR = 2.9, 95% CI: 1.23-6.62, *p* = 0.01). Children who had bacteremia were 10 times more likely to die than those who had no bacteremia (aRR = 10.0, 95% CI: 3.62-29.01, *p* = 0.01). Children who had HIV were 4.8 times at risk of dying compared with participants who had no HIV (aRR = 4.8, 95% CI: 1.42-16.30, *p* < 0.01). Children who had TB were 4.3 times more likely to die compared to participants who had no tuberculosis (aRR = 4.3, 95% CI: 1.28-14.49, *p* < 0.02). Children with shock had 60.9 times higher risk of death compared to participants who had no shock (aRR = 60.9, 95% CI: 9.05-410.28, *p* < 0.01).

## 4. Discussion

The proportion of mortality in our study population was 14.5% (39/338). This mortality of 14.5% is above the acceptable rate of less than 5% recommended by WHO.

The mortality of 14.5% meaning 1.5 death out of 10 or every 3 deaths out of 20 patients is higher compared to the mortality found in central and northern Uganda by Nabukeera-Barungi et al. [8] in Mulago National Referral Hospital and Nyeko et al. [7] in Lacor Hospital that showed a mortality of 9.8% and 11.9%, respectively. This could be explained by the study setting that is congested with many patients with nurse to patients' ratio of 1 to 26. Another possible reason for the difference in the study findings could be due to the variation in the study designs evidenced by the nested cohort in a randomized controlled trial, investigating the effect of probiotics on diarrhea among children admitted with SAM used the first study and the retrospective study design looking at the treatment outcome used in the second study.

However, this mortality of 14.5% is lower than 46% in study by Munthali et al. [12] in Zambia. The discrepancy in the study results could have risen due to the difference in the study designs employed in the two studies since the previous study was a retrospective quantitative review of hospital-based records whereas the present study is a cohort study. With the previous study utilizing only hospital records, there is a possibility of over exaggeration of mortality rate due to inaccuracy during the entry of information in the hospital database. Another reason for the lower mortality in the current study could be explained by the duration that took a period of only 3 months compared to a 5-year retrospective review. The finding of the current study is almost similar to the findings of a study done in Kenya by Gachau et al. [13] that revealed a proportion of 15.6%. The similarity of the study findings in the two studies can possibly be explained by the geographical areas where the two studies were conducted in that both studies were conducted in East Africa. Another reason for the similarity in the proportion could be because both studies were hospital-based studies.

In this study, it was found out that the risk of death was higher among males compared to females, and the majority of death occurred below 72 hours of hospital stay. The high mortality observed among males in this study could be explained the high number of admission of males compared to females (208 males versus 130 females). The high mortality during stabilization phase could be explained either by the poor response of the body that has undergone reductive adaptation because of the malnutrition to the medical intervention or the poor quality of medical intervention during that phase. The finding in the current study was in line with the mortality trends by Tette et al. [14] in Ghana that reported high mortality in males (52.2%) compared to females (47.8%) and similar to other studies in Africa, but it differed from the study in Mali which reported significantly higher case fatality rates among girls. More so, in Zambia, it was found that the mortality pattern was higher in males than females, and the mortality at 24 hours, 48 hours, and 1 week of admission was 93.2%, 88.7, and 29%, respectively [12].

In this study, it was found out that shock was found to be an independent predictor of mortality. Study participants with shock were 60.9 folds more likely to die compared to those who never had shock. This result means that shock was an independent predictor among the study participants. Nevertheless, this high adjusted RR with a very wide confidence interval (aRR: 60.9, 95% CI: 9.05-410.28) should be considered with caution because of the large number of the comparison group. This high risk of death among study participants with shock might be explain by either a poor body response to the medical interventions or an inadequate clinical or paraclinical monitoring of those patients whose metabolism had completely changed because of the reductive adaptation. However, the result of the present study is in line with the result of an institution-based retrospective cohort study in Ethiopia by Wagnew et al. [15] among under five children with severe acute malnutrition which revealed that children with shock had 8 times more risk of death compared to those without shock (AHR: 7.9, 95% CI: 3.7-16.7).

More so, the finding of the present study is also in accordance with the finding of Gebremichael et al. [16] which showed that children with shock were more likely to die than their counterparts though shock was significant only at bivariate analysis (CHR: 6.0, 95% CI: 1.77, 20.45) but was not found to be significant at the multivariable Cox regression analysis (AHR: 2, 95% CI: 0.51, 7.84). The result of this study is further supported by a study done by Kumar et al. [17] who found that children with fatal outcome were 11.29 times more likely to have shock (*p* = 0.001). Contrary to this study, Nabukeera-Barungi et al. [8] in their study done at Mulago National Referral Hospital excluded all the children who had shock from their study, and yet, there is a possibility that they could have found some association between mortality and shock as it has been the case in the present study.

This study revealed that anemia was one of the comorbidities with 2.9 times increased likelihood of mortality compared to those with no anemia. This could be explained either by a poor body response to transfusion or by a delayed transfusion due to the large number of patients to attend to. This finding coincides with that of Wagnew et al. [15] in Ethiopia in which anemia was an independent predictor of mortality among children with SAM (AHR = 2.3, 95% CI: 1.2-4.5). However, Savadogo et al. [18] in Burkina Faso did not find a difference in mortality among severely malnourished children with anemia (12.4%) and those without anemia (22.2%, *p* = 0.12). The latter study was in disagreement with other studies as it argued that anemia was not statistically associated with mortality of the children admitted with SAM. This could be explained by the study setting and the different medical interventions.

HIV positive study participants were 4.8 times more likely to die compared to HIV negative participants. This could be explained by an increased susceptibility to opportunistic infections and other comorbidities that could worsen their prognosis. This finding was in line with that of Nyeko et al. [7] in northern Uganda that revealed a significant association between HIV and mortality among children with SAM, 18.6% versus 9.6% in children HIV negative with OR = 3.1 (1.307-7.292), but it differed from that of Nabukeera-Barungi et al. [8] that found an association between HIV status of the children and mortality on bivariate analysis that disappeared on multivariate analysis after adjusting for age and sex of the children. More so, the result of the current study was in line with that of Munthali et al. [12] in Zambia who found that HIV-infected children were 1.8 times more likely to die than those who were HIV negative (OR = 1.8, 95% CI 1.6–2.0, *p* < 0.001). The geographical regions and the study designs would explain the disparities.

The study participants with tuberculosis had 4.3 times risk of death than those without tuberculosis. This could be explained by a poor host defense against tuberculosis, increasing the risk of death among those patients. This result differs from that of Wagnew et al. [15] in Ethiopia that revealed no significant statistical association between tuberculosis and mortality among the study participants. Moreover, Gebremichael et al. [16] retrospectively reviewed records of children with SAM admitted in three selected hospitals from Ethiopia, and they found 5.3% of the study participants to be having tuberculosis, but they never went ahead to do regression analysis to establish whether there was any association between tuberculosis and mortality of the study participants.

In the present study, study participants with bacteremia had 3.8 folds the risk of death compared to their counter parts who never had bacteremia. This could be explained by the impairment of the immune system due to malnutrition, causing bacterial overgrowth and infection that increased the morbi-mortality among those children. This result is in disagreement with the results of Nabukeera et al. that showed an association between bacteremia and mortality in bivariate analysis among study participants (OR 2.23, 95% CI 1.18–4.24, *p* = 0.01) but not on multivariate analysis (OR 0.3, 95% CI 0.04–2.24, *p* = 0.20). However, this result is in line with Maitland et al. [19] who demonstrated that bacteremia complicated 27% of all death and bacteremia had a significant *p* value of 0.03. On the other hand, the result of the present study is in agreement with Roy et al. [20] in Dhaka Hospital who revealed that participants with clinical septicaemia were 8.8 times the odds of death than study participants who never had septicaemia (AOR 8.8, 95% CI 3.7–21.1, *p* = 0.01). The magnitude of risk of death of study participants with bacteremia in the present study is low compared to the previous study, and the difference could be explained by the study design, the previous study being a case-control study meanwhile the present study was a prospective cohort study.

## 5. Conclusions

The proportion of death among children under 5 years admitted in the nutritional unit of Jinja Regional Referral Hospital was 14.5% and higher than the national average.

The most important comorbidities associated with mortality were anemia, bacteremia, HIV, TB, and shock. Based on the findings, it is recommended that health care providers should encourage caregivers of children with SAM to seek for care in time, particularly those with comorbidities.

The clinicians are recommended to follow-up closely patients with severe acute malnutrition and to focus on the critical comorbidities identified and also avoid unnecessary medical interventions.

## Figures and Tables

**Table 1:** Factors associated with mortality among children below 5 years with severe acute malnutrition in JRRH.

Factor	Mortality		cRR (95% CI)	*p*	aRR (95% CI)	*p*
	Yes, *n* = 49	No, *n* = 289				
Sex of child						
Males	39 (18.75)	169 (81.25)	1.0			
Females	10 (7.69)	120 (92.31)	0.4 (0.17-0.75)	0.01	0.39 (0.17-0.87)	0.02
Age of child in months						
≤ 12	14 (13.08)	93 (86.92)	1.0		—	—
13-24	26 (14.53)	153 (85.47)	1.1 (0.56-2.27)	0.73	—	—
> 24	9 (17.31)	43 (82.69)	1.3 (3.55-3.46)	0.48	—	—
Immunization status						
Complete	25 (14.12)	152 (85.88)	1.0		—	—
No/incomplete	24 (14.91)	137 (85.09)	1.0 (0.58-1.95)	0.84	—	—
Duration of admission						
< 24 hours	4 (80.00)	1 (20.00)	1.0			
24-71 hours	10 (83.33)	2 (16.67)	1.2 (0.08-17.97)	0.87	1.73 (0.11-27.20)	0.70
72 hours +	35 (10.90)	286 (89.10)	0.04 (0.00-0.28)	0.01	0.04 (0.00-0.40)	0.01
Exclusive breastfeeding						
Yes	14 (12.84)	95 (87.16)	1.0			
No	35 (15.28)	95 (87.16)	1.2 (0.62-2.38)	0.55	—	—
Caregiver's relationship to child						
Mother	36 (15.19)	201 (84.81)	1.0			
Father	4 (14.29)	24 (85.71)	0.9 (0.30-2.84)	0.90	—	—
Grandparent	7 (12.73)	48 (87.27)	0.8 (0.34-1.94)	0.64	—	—
Others	2 (11.11)	16 (88.89)	0.6 (0.15-3.16)	0.64	—	—
Age of caregiver in years						
< 20 years	2 (8.00)	23 (92.00)	1.0		—	—
20-29 years	30 (18.18)	81 (.82)	2.5 (0.57.11.43)	0.22	—	—
30-49 years	14 (11.57)	107 (88.43)	1.5 (0.31-7.07)	0.61	—	—
50+ years	3 (11.11)	24 (88.89)	1.4 (0.21-9.40)	0.71	—	—
Residence						
Urban	5 (7.81)	59 (92.19)	1.0		—	—
Rural	44 (16.06)	230 (83.94)	2.3 (0.85-5.94)	0.10	—	—
Highest education level						
No formal	15 (18.99)	64 (81.01)	1.0			
Primary	29 (15.43)	159 (84.57)	0.8 (0.39-1.54)	0.48	1.12 (0.49-2.57)	0.79
Secondary +	5 (7.04)	66 (92.96)	0.3 (0.11-0.94)	0.04	0.38 (0.10-1.41)	0.15
Occupation						
Peasant	8 (14.29)	48 (85.71)	1.0			
Business	35 (17.07)	170 (82.93)	1.2 (0.53-2.83)	0.62	—	—
Civil servant/formal	6 (7.79)	71 (92.21)	0.5 (0.16-1.55)	0.24	—	—
Average monthly income of caregiver (Ugx)						
< 100,000 Ushs	43 (16.6)	216 (83.4)	1.0			
100,000 + Ushs	6 (7.59)	73 (92.41)	0.4 (0.16-1.00)	0.05	0.62 (0.23-1.65)	0.34

**Table 2:** Comorbidities associated with mortality among children below 5 years with severe acute malnutrition in JRRH.

Complication	Mortality		cRR (95% CI)	*p*	aRR (95% CI)	*p*
	Yes	No				
Dehydration						
No	33 (17.62)	173 (82.38)	1.0			
Yes	12 (9.38)	116 (90.63)	0.48 (0.24-0.96)	0.04	0.8 (0.31-2.20)	0.71
Pneumonia						
No	29 (13.74)	182 (86.26)	1.0		—	—
Yes	20 (15.75)	105 (84.25)	1.17 (0.63-2.18)	0.61	—	—
Malaria						
No	34 (13.55)	217 (84.45)	1.0		—	—
Yes	15 (17.24)	72 (82.76)	1.3 (0.68-2.68)	0.40	—	—
Acute diarrhea						
No	44 (16.6)	221 (83.4)	1.0			
Yes	5 (6.85)	68 (93.15)	0.4 (0.14-0.96)	0.04	0.2 (0.04-0.88)	0.03
Anemia						
No	32 (11.55)	245 (88.45)	1.0			
Yes	17 (27.87)	44 (72.13)	2.9 (1.5-5.8)	0.01	2.9 (1.23-6.62)	0.01
Bacteremia						
No	36 (11.43)	279 (88.57)	1.0			
Yes	13 (56.52)	10 (43.48)	10.1 (4.11-24.64)	0.01	10.0 (3.62-29.01)	0.01
HIV +						
No	42 (13.21)	279 (86.79)	1.0			
Yes	7 (35.0)	13 (65.0)	3.5 (1.33-9.37)	0.01	4.8 (1.42-16.30)	0.01
TB						
No	42 (13.08)	279 (86.92)	1.0			
Yes	7 (41.21)	10 (58.82)	4.6 (1.67-12.88)	0.01	4.3 (1.28-14.49)	0.02
Electrolyte imbalance						
No	48 (14.81)	276 (85.19)	1.0		—	—
Yes	1 (7.14)	13 (92.86)	0.4 (0.56-3.45)	0.44	—	—
Shock						
No	39 (11.96)	287 (88.04)	1.0			
Yes	10 (83.33)	2 (16.67)	36.7 (7.77-174.15)	0.01	60.9 (9.05-410.28)	0.01
Hypothermia						
No	46 (13.86)	286 (86.14)	1.0			
Yes	3 (50.00)	3 (50.00)	6.2 (1.21-31.74)	0.03	0.9 (0.09-8.80)	0.94
Cardiac failure						
No	49 (14.63)	286 (85.37)	—		—	—
Yes	0 (0.00)	3 (100.00)	—		—	—

cRR: crude relative risk; aRR: adjusted relative risk; CI: confidence interval; *p* value is significant at 0.05.

## Data Availability

The data used to obtain the findings is available from the corresponding author DB and the author FKS on a reasonable request.

## Acronyms

F75: Feeding formula 75
F100: Feeding formula 100
IMAM: Integrated Management of Acute Malnutrition
ITC: Inpatient Therapeutic Care
NR: Nutritional Rehabilitation
OTC: Outpatient Therapeutic Care
ReSoMal: Rehydration Solution for Malnutrition
RRH: Regional Referral Hospital
SAM: Severe acute malnutrition
SDG: Sustainable Development Goal
SFP: Supplementary Feeding Programme
TB: Tuberculosis
UNICEF: United Nations International Children's Emergency Fund
UTI: Urinary Tract Infection
WHO: World Health Organization.
